# Debonding mechanism of zirconia and lithium disilicate resin cemented to dentin

**DOI:** 10.1080/23337931.2018.1561188

**Published:** 2019-01-24

**Authors:** Mina Aker Sagen, Ketil Kvam, Eystein Ivar Ruyter, Hans Jacob Rønold

**Affiliations:** a Institute of Clinical Dentistry, University of Oslo, Oslo, Norway;; b NIOM, Oslo, Norway

**Keywords:** Zirconia, ceramics, resin cement

## Abstract

To evaluate debonding mechanism of zirconia and lithium disilicate cemented to dentin mimicking what could occur in a clinical setting. A null hypothesis of no difference in tensile bond strength between groups of zirconia and lithium disilicate cemented with resin cements was also tested. Zirconia rods (*n* = 100) were randomly assigned to two different surface treatment groups; air borne particle abrasion and hot etching by potassium hydrogen difluoride (KHF_2_). Lithium disilicate rods (*n* = 50) were surface etched by hydrofluoric acid (HF). Five different dual cure resin cements were used for cementing rods to bovine dentin. Ten rods of each test group were cemented with each cement. Test specimens were thermocycled before tensile bond strength testing. Fracture morphology was visualized by light microscope. Mean surface roughness (Sa value) was calculated for randomly selected rods. Cohesive fracture in cement was the most frequent observed fracture morphology. Combination of adhesive and cohesive fractures were second most common. Fracture characterized as an adhesive between rod and cement was not observed for KHF_2_ etched zirconia. Highest mean tensile bond strength was observed when cementing air borne particle abraded zirconia with Variolink Esthetic (Ivoclar Vivadent). All surface treatments resulted in Sa values that were significant different from each other. The number of cohesive cement fractures observed suggested that the cement was the weakest link in bonding of zirconia and lithium disilicate.

## Introduction

Zirconia has become one of the most used ceramic in prosthetic dentistry the last decades [1].

The material has a high flexural strength [2] due to its crystal content and transformation toughening from crystal transformation [3]. These characteristics make it appropriate for use as both core material in bi-layered restorations or as monolithical restorations with smaller dimension [4].

Despite excellent mechanical properties of zirconia there are complications related to clinical use. Loss of retention of tooth supported crowns is reported as one of the most frequent technical complication. Many approaches have been studied with the aim to increase bond strength between resin cement and zirconia [5]. Tribochemical silica coating, plasma spraying, selective infiltration technique, hot etching and different lasers have been investigated [6]. The results varied when it came to both tensile and share bond strength in laboratory tests, and storage in water or thermocycling showed low predictability of a stable bond [7].

Air borne particle abrasion using particles of aluminum oxide, diamond or boron nitride is the most used surface treatment [6]. This technique is often combined with 10-methacryloyloxydecyl dihydrogen phosphate (10-MDP) containing primer to create a chemical bond [2]. Air borne particle abrasion of zirconia surface has shown phase transformation from tetragonal to cubic and monoclinic crystal structure due to temperature changes [8]. This might reduce flexural strength and potentially lead to fracture [9]. Recommendations from different producers regarding air borne particle abrasion vary, both in particle size and pressure, even if it should be performed as a surface treatment because of potential risks.

High crystallinity of zirconia and lack of glass phase makes the material resistant to etching by hydrofluoric acid (HF). This is in contrast to lithium disilicate, where etching by HF establish micromechanical and chemical bond to silanoles and resin cement [6].

An alternative method of surface etching of zirconia was studied by Ruyter et al. [8]. High share bond strength was observed when fluoride compounds were used for hot etching, and quantitative analysis detected low volume fracture of monoclinic crystals in the surface. SEM images of the surface after testing showed cement partly remaining on zirconia, indicating a strong bond between the etched surface and cement [8].

When cementing ceramic restorations using resin cement, the tooth substance is often pretreated by acidic etch and adhesive components, either as multiple or single step. The pretreatment creates mechanical interlocking and chemical bond between tooth substance and adhesive [10].

In a clinical setting, loosening of the restoration may occur in the weakest part, which represents the bond strength. This could be the cement – restoration bond, in the cement – tooth structure bond or in the cement itself.

The aim of the present study was to evaluate debonding mechanism of zirconia and lithium disilicate cemented to dentin mimicking what could occur in a clinical setting, and to test the null hypothesis that no difference in tensile bond strength between groups of zirconia and lithium disilicate cemented with resin cements would be found.

## Materials and methods

### Preparation of specimen

Bovine mandibular incisors (*n* = 150) were extracted (from bovine cadaver, 4–6 years old, Nortura), cut 2 cm length and embedded in epoxy resin (EpoFix, Struers) with buccal surface exposed. Embedded teeth were ground at DP-U2 with rotating 500-grit silicon carbide paper (Struers, Denmark) under water until 5 × 5 mm dentin surface was obtained and further stored in distilled water.

**Figure 1.**
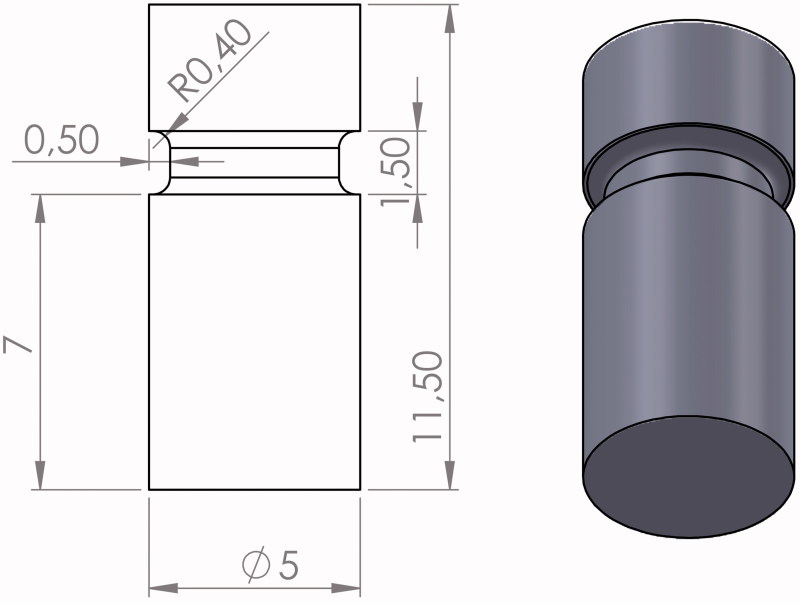
Design of ceramic rod. The illustration shows the dimensions in mm and a copy of the computer aided design (CAD).

Circular zirconia (*n* = 100, Starceram Z, H.C. Starck Ceramics GmbH, Germany) and lithium disilicate (*n* = 50, IPS e.max CAD, Ivoclar Vivadent, Lichtensein) rods with diameter of 5 mm and length of 11.5 mm were produced by CAD/CAM technique. Rods were produced with a notch in the circumference (Figure 1) facilitating the grip during tensile testing.

One end of the rods was ground with 500 grit silicon carbide sandpaper under water to reflect use of a fine bur in a clinical situation [11,12], and to obtain uniform surface roughness. There after cleaned with a dental steam cleaner (Steamer X3, Amann Girrbach, Austria) and thoroughly air-dried.

### Surface treatment of zirconia and lithium disilicate rods

Zirconia rods were randomly assigned to two different surface treatment groups (*n* = 50 each group), lithium disilicate rods (*n* = 50) formed one group. The groups were:

- Zir-A: zirconia, air borne particle abraded, 50 µm aluminum oxide (Al_2_O_3_, Korox)
- Zir-E: zirconia, etched by potassium hydrogen difluoride (KHF_2_)
- LDS: lithium disilicate etched by 4.5% hydrofluoric acid (HF)

Five resin based cements were used for cementation of the rods (*n* = 10 of each group with each cement).

#### Hot etching procedure

KHF_2_ was ground to fine powder using a mortar and inflicted equally on the bonding surface of zirconia rods. Thereafter rods were heated in a precalibrated furnace (Jelenko, acc-therm II 2000, NY-USA) for 10 min at 280 °C for the KHF_2_ to melt. After cooling, rods were thoroughly steam cleaned and ultrasonically cleaned in distilled water for 15 min. Finally, they were air-dried.

#### Air borne particle abrasion

Zirconia rods were air borne particle abraded at 2.5 bar for 10 s. The nozzle was kept perpendicular to the zirconia surface at 10 mm distance. Rods were air steamed and ultrasonically cleaned in distilled water for 15 min before thoroughly air-dried.

#### Hydrofluoric acid

The bonding surface of lithium disilicate glass ceramic rods were etched with hydrofluoric acid (HF 4.5%, IPS Ceramic Etching Gel, Ivoclar Vivadent) for 20 s, cleaned by running water >20 s and thoroughly air-dried.

### Surface evaluation

The surface on randomly selected rods was studies in scanning electron microscope (Hitachi Analytical TableTop Microscope/Benchtop SEM TM3030), with energy dispersive spectroscopy, EDS. Surface roughness was measured using a confocal microscope (Sensofar S neox). Mean surface roughness (Sa value) was calculated for randomly selected rods [13].

### Cementation

Five different dual cure resin cements were used for cementing rods to bovine dentin; Multilink Automix (Ivoclar Vivadent), Variolink Esthetic (Ivoclar Vivadent), Panavia F2.0 (Kuraray Noritake Dental), Duo-Link (Bisco) RelyX Unicem (3 M ESPE) (Table 1).

**Table 1.** Materials used for cementing.

Cement	Manufacturer	Adhesive	Manufacturer	Ceramic primer	Manufacturer
Variolink Esthetic	Ivoclar Vivadent	Adhese	Ivoclar Vivadent	Monobond Plus	Ivoclar Vivadent
Multilink Automix	Ivoclar Vivadent	Multilink primer A & B	Ivoclar Vivadent	Monobond Plus	Ivoclar Vivadent
Panavia F2.0	Kuraray Noritake Dental	ED primer 2 A & B	Kuraray Noritake Dental	Clearfil Ceramic Primer Plus, Clearfil SE Bond Primer, Porcelain Bond Activator	Kuraray Noritake Dental
Duo-Link	Bisco	All-Bond 2 primer A & B, Pre-Bond Resin, D/E Resin	Bisco	Z-prime Plus, Bis-silane	Bisco
RelyX Unicem	3M			Bis-silane	Bisco

Cementation was performed according to producers’ manual and primer was applied when recommended (Table 1).

Ten rods from each of the three groups; KHF_2_ etched zirconia, air borne particle abraded zirconia and HF etched lithium disilicate, were cemented by each cement.

Dentine was cleaned using pumice powder dispensed in water prior to cementation.

After placing the rods onto dentin, a standardized 882 g seating load was applied by a cementation jig. Excess cement was removed using quick stick micro-brush before light curing 20 s each from 4 directions.

All specimens were kept dry at room temperature for 15 min following cementation and thereafter immersed in 37°C distilled water for 24 h.

Specimens were sandblasted using Al_2_O_3_ to remove cement remnant outside the rods and evaluated by light microscopy. Test units were thermocycled 5000 cycles in 5 °C and 55 °C water baths.

### Tensile bond strength testing

**Figure 2.**
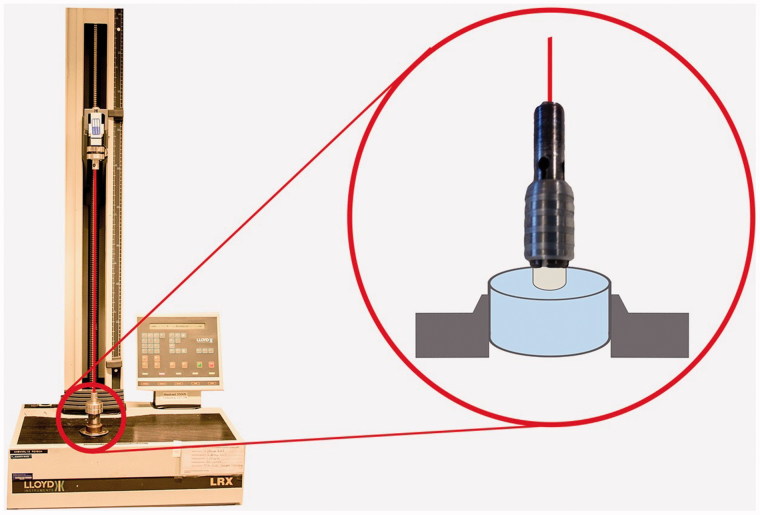
Experimental design of tensile bond strength test. A metallic jig enclosed the ceramic rod at the notch in the circumference for adequate grip. The rod was cemented onto the dentin surface of bovine tooth embedded in epoxy resin.

Specimens were mounted in a universal mechanical test machine (Lloyd LRX, Lloyd Instruments Ltd, Leicester, UK). Tensile force was applied until break using a centered wire with a cross head speed of 1 mm/min. Figure 2 illustrates the experimental design of tensile bond strength test. Tension force (N) at break was recorded and tensile bond strength (MPa) calculated in Nexygen DF Force Measuring Software.

### Fracture characterization

Rods and dentin were studied in light microscope (American Optical Stereo Star/Zoom, model 570, American Optical Corporation, Buffalo NY, USA. Magnification 10X–63X) for visualizing fracture morphology.

Fractures were classified in to 5 different types: (1) adhesive failure between cement and rod, (2) adhesive failure between cement and dentin, (3) cohesive in cement (4) cohesive failure in dentin, (5) combination of adhesive and cohesive failure.

### Statistical analysis

Microsoft Excel (version 14.2.3) was used for calculating mean tensile bond strength and standard deviation. Komogorov–Smirnov was used for calculation of normality and differences among groups were evaluated using ANOVA tests followed by Tukey’s HSD test. Evaluations were done (1) among rod materials for each cement and (2) among cements for each rod material. *p* < .05 was regarded as statistical significant different.

## Results

### Fracture morphology

**Figure 3.**
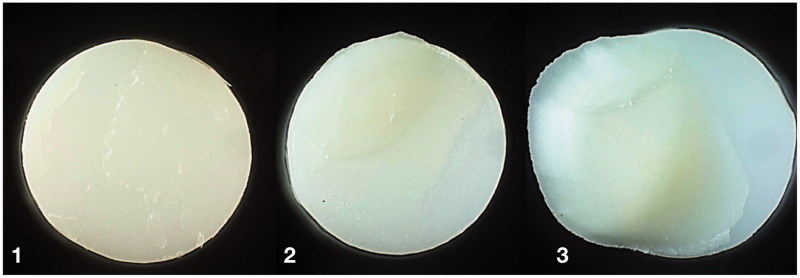
Examples of fracture morphology observed in light microscope (diameter 5 mm). 1: combination of cohesive fracture in cement and adhesive fracture between cement-zirconia; 2: combination of cohesive fracture in dentin and adhesive fracture between cement-dentin and cement-zirconia; 3: combination of cohesive fracture in dentin and cement, and adhesive fracture cement-zirconia.

Cohesive fracture in cement was the most common fracture morphology visualized by light microscope, as presented in Table 2. Duo-Link cement showed exclusively cohesive fractures in cement, regardless of test group. Combination of adhesive and cohesive fractures were second most common. Figure 3 show examples of fracture morphology observed in light microscope. Fracture characterized as adhesive between rod and cement was not observed for KHF_2_ etched zirconia.

**Table 2.** Fracture characterization.

	Adhesive						Cohesive								
Fracture type	Dentin-cement			Rod-cement			Dentin			Cement			Combination		
Cement/material	Zir A	Zir E	LDS	Zir A	Zir E	LDS	Zir A	Zir E	LDS	Zir A	Zir E	LDS	Zir A	Zir E	LDS
Multilink Automix			1			2	2	2		1		2	7	8	5
Variolink Esthetic	1		4				3			1	10	2	5		4
Panavia F2.0				2						6	9	10	2	1	
Duo-Link										10	10	10			
RelyX Unicem	5	1						1			2	8	5	6	2

The table show number of adhesive, cohesive, and combined fractures for each material and cement. Rods and dentin were studied in light microscope for visualizing fracture morphology. Fractures were classified into 5 different types based on the type for 2/3 of the surface. Fracture was classified as combined if less than 2/3 was of one specific type.

Zir A: air borne particle abraded zirconia; Zir E: KHF2 etched zirconia; LDS: hydrofluorid acid etched lithium disilicate.

**Figure 4.**
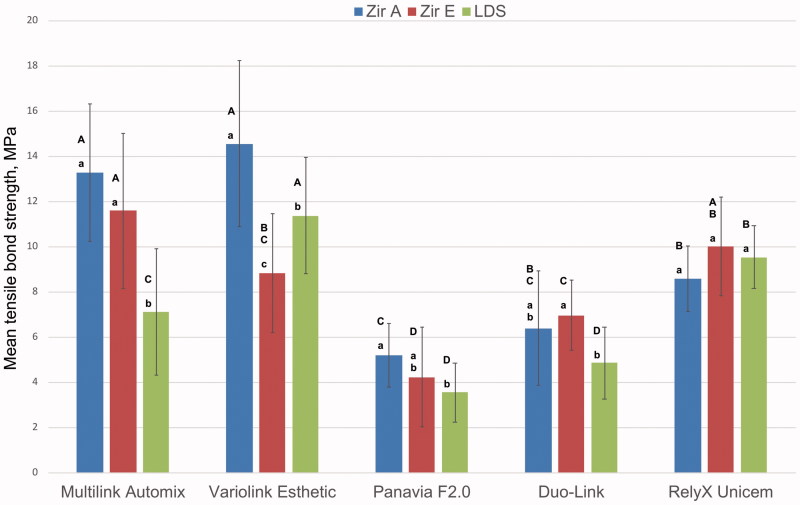
Mean tensile bond strength and standard deviation. Zir A: air borne particle abraded zirconia; Zir E: KHF_2_ etched zirconia; LDS: hydrofluorid acid etched lithium disilicate. Different lowercase letters illustrate significant difference (*p* < .05) between Zir A, Zir E, and LDS for each cement. Different uppercase letters illustrate significant differences (*p* < .05) between cements for each rod material.

Adhesive fracture between cement and dentin and cohesive fracture in dentin was observed for Multilink Automix, Variolink Esthetic and RelyX Unicem. This was also the cements with the highest tensile bond strength, as shown in Figure 4.

### Tensile bond strength

Mean tensile bond strength and standard deviation for the three test groups cemented with different dual cure resin cements are illustrated in Figure 4. Results of ANOVA and Tukey`s HSD tests calculated for differences between test rods for each cement and between cements for each rod material are also given in Figure 4. Highest mean tensile bond strength was observed when cementing air borne particle abraded zirconia with Variolink Esthetic and Multilink Automix cement. The lowest bond strengths were obtained with Panavia F2.0 and Duo-Link. There were no differences regarding the effects of the different surface treatments of the zirconia rods for all cements, except for Variolink Esthetic where air borne particle abraded zirconia showed higher bond strength. Compared to both zirconia rod types, lithium disilicate rods had lower or similar mean tensile bond strength to all cements except Variolink Esthetic.

### Surface evaluation

**Figure 5.**
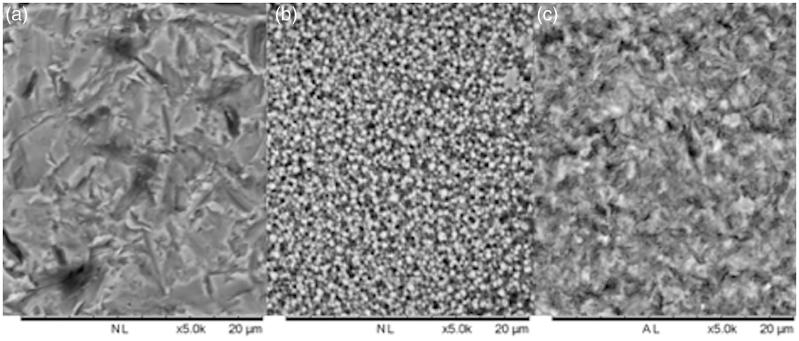
Representative SEM images of air borne particle abraded zirconia (a), KHF_2_ etched zirconia (b), and hydrofluoric acid etched lithium disilicate (c). Bar represents 20 μm.

Sa value after surface treatment of randomly selected rods were measured using a confocal microscope. As presented in Table 3, air borne particle abraded zirconia had the highest Sa value. All surface treatments resulted in Sa values that were significant different from each other. The marked differences in surface morphology of the three test groups are visualized in SEM images (Figure 5).

**Table 3.** Mean surface roughness (Sa) measured in nanometer and statistical comparison between the groups.

Parameter	Zir A	Zir E	LDS	Zir A/ Zir E	Zir E/LDS	Zir A/LDS
Sa	534–592	127–131	184–255	*p* < .01	*p* < .01	*p* < .01

Zir A: air borne particle abraded zirconia, Zir E: KHF_2_ etched zirconia, LDS: hydrofluoric acid etched lithium disilicate.

## Discussion

The aim of the present study was to evaluate debonding mechanism of zirconia and lithium disilicate cemented to dentin to mimic what can occur in a clinical setting. A null hypothesis of no difference in tensile bond strength between groups of zirconia and lithium disilicate cemented with resin cements was also tested.

Five dual cure resin cements were used for cementing rods of zirconia (KHF_2_ etched and air borne particle abraded) and lithium disilicate (HF etched).

Cohesive fracture in cement was the most frequent fracture morphology visualized by light microscope. Combinations of cohesive and adhesive fractures were second most common.

In a clinical setting loosening of restorations might be because of debonding between cement and dentin, between cement and ceramic or cohesive fracture in cement. Main focus of previous studies on zirconia has been on increasing bond strength between the ceramic and resin cement [6]. Ruyter et al. [8] found increased shear bond strength when zirconia was etched by KHF_2_ instead of air borne particle abraded. The authors report adhesive fractures with bonding agent partly remaining on zirconia. In the present study, no exclusive adhesive fractures between cement and KHF_2_ etched zirconia were observed, suggesting that other interfaces are important to increase bond strength. Melt etching creates a rough surface of zirconia grains which facilitates the micromechanical retention of coupling agent and luting cement. It is anticipated that by the treatment of zirconia with KHF_2_ the surface is fluoridated, which after steam and ultrasonic water treatment is hydrolyzed leaving active hydroxyl groups (OH^−^) [8]. Adhesive failure was only detected for two air borne particle abraded zirconia rods and two lithium disilicate rods, cemented with Panavia F2.0 and Multilink Automix respectively. Even though surface treatment of rods in the three different groups were performed as equal as possible, these findings might be explained by variation in micro mechanical and chemical surface properties.

The 10-MDP containing cement, Panavia F2.0, had the lowest tensile bond strength and was the cement with the highest number of cohesive fractures. The low value is in contrast to the previous study on 10-MDP containing cement [14]. 10-MDP is an acid functional monomer with two OH-groups bonded to phosphorous where pka_1_ value is 2.2 [15]. Primary chemical bonds to zirconia together with hydrogen bonds can be formed [16].

Multilink Automix was the only cement showing adhesive debonding to lithium disilicate. This was somewhat unexpected finding. Lithium disilicate etched with hydrofluoric acid and primed with silane containing primer is known for establishing micromechanical and chemical bond between resin cement and ceramic [4]. Adhesive fracture between cement and dentin and cohesive fracture in dentin were observed for cements with the highest mean tensile bond strength. This indicates that the bond of zirconia and lithium disilicate to these three resin cements is stronger than the bond between cement and tooth substance.

Combination of adhesive and cohesive fractures in dentin were also common for the three cements with the highest tensile bond strength, this specially applies for zirconia rods regardless of surface treatment. The bonding seemed to be nearly as strong as the inherent strength of the dentin, which must be regarded as the maximum bond strength.

All cements had cohesive fractures to different degree. These observations indicate that cements have different cohesive bond strength that will affect the retention of adhesive cemented restorations.

A thin and uniform cement layer is recommended to reduce shrinkage stresses during polymerization and loading failure of the ceramic [17]. However, there is no standardized procedure for cementing. In previous studies performed on bond strength of zirconia and lithium disilicate, different loading weight on the cement has been used, 50 N, 750 g, 15 N [8,18,19]. When cementing zirconia and lithium disilicate rods to dentin in this study, a standardized 882 g seating load was applied during light curing. Normally this should result in a uniform cement space for all specimens which will not influence the results.

Cements used today are mainly in the form of automix to ensure equal amount and even mix of components in the cement system. In the present study one cement, Panavia F2.0, was mixed by hand as recommended by the manufacturer. Panavia F2.0 showed the lowest mean tensile bond strength for all three ceramics. The mixing procedure could result in non-homogenous cement and contributed to the weak bond strength.

Micro roughness in the bonding surface of zirconia and lithium disilicate is necessary to establish good bond to resin cement [6]. In this study, surface roughness was created either by air borne particle abrasion or hot/cold etching. All treatments resulted in micro roughness that were significantly different from each other. Increased surface roughness implies a larger surface for bonding and a higher bond strength. Only a few adhesive fractures between cement and ceramic rods were observed. This indicated that other aspects than the surface of zirconia and lithium disilicate was important for the bond strength.

To obtain a clinical perspective in the present study, dentin was chosen as substrate for which zirconia and lithium disilicate were cemented to. Individual differences in dentin are detected in several studies [20] and can affect bonding mechanism and retention of restorations relying on adhesive cementation [21].

## Conclusion

The number of cohesive cement fractures observed in the present study suggested that the cement was the weakest link in bonding of ceramics. The null hypothesis of no difference in tensile bond strength between groups of zirconia and lithium disilicate cemented with resin cements was rejected for some combinations.
